# Absence of an Intron Splicing Silencer in Porcine *Smn1* Intron 7 Confers Immunity to the Exon Skipping Mutation in Human *SMN2*

**DOI:** 10.1371/journal.pone.0098841

**Published:** 2014-06-03

**Authors:** Thomas Koed Doktor, Lisbeth Dahl Schrøder, Henriette Skovgaard Andersen, Sabrina Brøner, Anna Kitewska, Charlotte Brandt Sørensen, Brage Storstein Andresen

**Affiliations:** 1 Department of Biochemistry and Molecular Biology, University of Southern Denmark, Odense M, Denmark; 2 Department of Biomedicine, Aarhus University, Aarhus C, Denmark; 3 Institute of Animal Reproduction and Food Research, Polish Academy of Sciences, Olsztyn, Poland; International Centre for Genetic Engineering and Biotechnology, Italy

## Abstract

Spinal Muscular Atrophy is caused by homozygous loss of *SMN1*. All patients retain at least one copy of *SMN2* which produces an identical protein but at lower levels due to a silent mutation in exon 7 which results in predominant exclusion of the exon. Therapies targeting the splicing of *SMN2* exon 7 have been in development for several years, and their efficacy has been measured using either in vitro cellular assays or in vivo small animal models such as mice. In this study we evaluated the potential for constructing a mini-pig animal model by introducing minimal changes in the endogenous porcine *Smn1* gene to maintain the native genomic structure and regulation. We found that while a *Smn2*-like mutation can be introduced in the porcine *Smn1* gene and can diminish the function of the ESE, it would not recapitulate the splicing pattern seen in human *SMN2* due to absence of a functional ISS immediately downstream of exon 7. We investigated the ISS region and show here that the porcine ISS is inactive due to disruption of a proximal hnRNP A1 binding site, while a distal hnRNP A1 binding site remains functional but is unable to maintain the functionality of the ISS as a whole.

## Introduction

The Spinal Muscular Atrophies (SMA) is a phenotypically diverse but genetically very similar group, in that the diseases are all caused by homozygous loss of the *SMN1* gene [1]. The disease modifier gene, *SMN2*, determines to some extent the phenotype of the affected individual and is unique to the hominid line [2]–[4]. As such, SMA caused by reduced amounts of SMN protein is a disease unique to humans and the study of animal models is therefore restricted to transgenic animals. Of these, mouse models have been used extensively in the past [5]–[8], but the metabolic and physiological differences between humans and mice are limiting the potential of the mouse model for evaluation of drug candidates and studying the molecular pathology of the disease in detail. Several metabolic and physiological symptoms have been described in mouse models, which are either rare or only observed in very severe human cases [9] or more likely explained by the genetic background of the particular model [10]. The pig is in many ways a better model of human biology and mini-pigs are especially good models since they grow to app. human size and weight as adults. Pigs are known to be more genetically similar to humans than mice are [11], and their metabolism as well as physiology more close to ours than the mice's. For these reasons we set out to evaluate the potential of constructing a mini-pig animal model of SMA in order to facilitate improved drug candidate testing and studies of disease pathology. Furthermore, a mini-pig model would be extremely valuable in determining the potential of stem cell treatments as the central nervous system (CNS) of pigs is very similar to the human CNS. An SMA pig model would therefore be relevant as an animal model for not only SMA but also other motor neuron diseases or in the case of traumatic injury to the motor neurons in the spinal cord.

The role of *SMN2* in humans is unclear in the population as a whole, but in SMA patients *SMN2* serves an important function as the remaining SMN expressing gene. It fails to completely compensate for the loss of *SMN1* due to aberrant splicing of exon 7 which leads to the production of predominantly truncated transcripts and a corresponding decrease in the amounts of functional protein [12]–[14]. As *SMN2* is present in all SMA patients it has been extensively studied and serves as a drug target for drugs that specifically correct splicing of exon 7 and thereby increases amounts of functional SMN protein [15]–[17]. As such, large animal models where broader effects of both early and late treatment can be carefully examined are becoming increasingly relevant. In particular, the bioavailability and therapeutic potential of drug candidates are more easily studied in animal models that more closely resemble the physiology and metabolism of humans. Transgenic models which have been generated through a knock-out/knock-in approach can potentially display pathologies unrelated to the trans-gene itself, but as a consequence of gene disruption caused by the insertion. This was recently reported in the widely used Tg(*SMN2*)89Ahmb mouse model of SMA [10].

In order to construct a transgenic pig which resembles the human SMA genotype as closely as possible we chose to study the potential in converting the endogenous pig *Smn1* to that of a human *SMN2* and in the process changing as little as possible in the endogenous gene.

The aberrant splicing of human *SMN2* exon 7 is caused by the loss of an exonic splicing enhancer (ESE) due to a +6C>T transition in *SMN2* exon 7 relative to *SMN1* exon 7, leading to loss of binding of SRSF1 and increased binding of hnRNP A1 due to strengthening of pre-existing exonic splicing silencer (ESS) motifs [12], [14], [18], [19]. In humans, the active ESE motif is altered from CAGACAA to an inactive TAGACAA motif in *SMN2*, but in pigs the ESE motif is only slightly altered to CAAACAA in the wild type *Smn1*. This poses the question of whether or not this sequence constitutes an active ESE and if a single *Smn2*-like +6C>T mutation in porcine *Smn1* exon 7 can disrupt the function and result in a porcine *Smn2*-like gene.

## Results

We began by sequencing the Yucatan mini-pig *Smn1* gene from genomic DNA by designing primers to amplify individual exons based on publicly available data as well as larger parts of the intronic regions surrounding exon 7 which were not publicly available at the time. Additionally, we performed 5′RACE and 3′RACE in order to validate previous assignment of exons and UTR regions. The resulting Yucatan *Smn1* gene sequence has been submitted to the GenBank sequence database under accession number KF585502. Contrary to previously published data [20], but in line with the recently published Duroc porcine genome from Ensembl (Sscrofa9), we found that porcine *Smn1* is composed of not 9 exons but instead 10, and that the last two exons are located much further downstream from exon 7 than in the human *SMN1* and *SMN2* genes.

**Figure 1:**
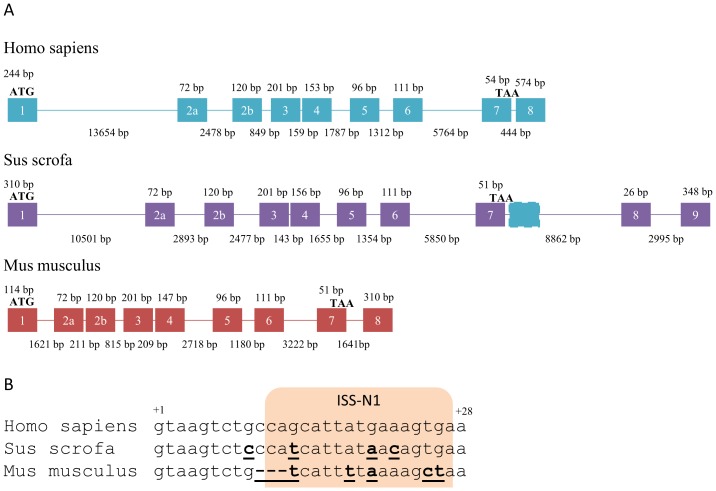
Genomic structure of SMN1 genes in humans, pigs and mice. **A**) *SMN1* pre-mRNA transcripts expressed in humans, pigs and mice. Exons included in transcripts are numbered according to historical nomenclature. Presence of pseudoexons in processed introns are indicated in dashed outline and coloured according to the species expressing transcripts including these exons. Introns are drawn to scale and indicated as lines, exons are not drawn to scale and are indicated as boxes. Start-codon is indicated by ATG and stop-codon by TAA. **B**) The start-sequence of intron 7 in humans, pigs and mice. Bases that differ from the human sequence are indicated in bold underline. The location of the human ISS is indicated in shade.

In humans, exon 7 is followed by a 444 bp long intron but in other animals, such as the mouse, intron 7 is much longer and the final exon, exon 8, is completely different from the human sequence. In pigs, however, exon 8 is a small 26 bp long exon located 8.9 kb downstream from exon 7 and is then followed by a 3 kb long intron 8 after which the porcine *Smn1* exon 9 begins (Fig. 1A). Despite the significant difference in intron lengths, porcine exon 9 and murine exon 8 seem to be homologous. One striking observation though, is the overall similarity between human *SMN1*/*SMN2* and porcine *Smn1* in terms of exon-intron structure, with the exception of the 3′ end of the transcripts, while the intron lengths are very different in murine *Smn1*.

It is interesting to note that the natural stop-codon of porcine *Smn1* is located in the third to last exon, but successfully escapes the NMD pathway (reviewed in [21]) as the stop-codon is within 50 bp from the last exon-exon junction. It is tempting to speculate on the mechanism by which the small exon 8 arose in pigs and whether or not it was ever used as an exon in other species such as mouse.

In addition to the endogenous *Smn1* gene, we also identified *Smn1* sequences derived from at least two separately processed pseudogenes, likely stemming from insertion of reverse transcribed mature mRNA (Fig. S1). Subsequent BLAST searches [22] revealed the location of these two pseudogenes on chromosome 2 and 13. We did not find evidence for expression of transcripts from these pseudogenes, and due to accumulated mutations it is doubtful that they would lead to production of functional protein if they were transcribed.

**Figure 2:**
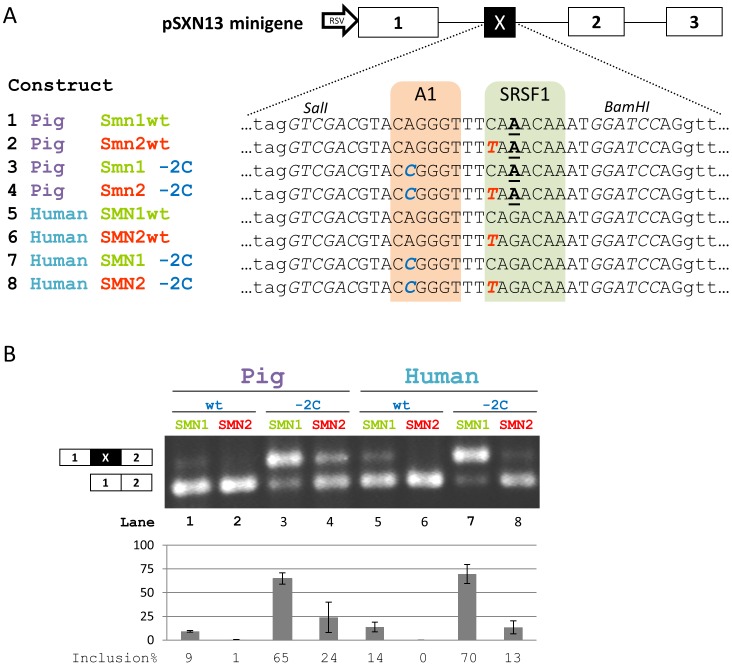
Splicing analysis of pSXN13 minigenes. **A**) Summary of the pSXN13 minigene and the mutations introduced in the different constructs. The hnRNP A1 binding sites within ISS-N1 have been indicated in dashed outline. Capitals indicate exonic bases. Bold italic bases in blue indicate introduced mutations. The +6C>T mutation is indicated in bold italic red. The +8G>A mutation in pigs is indicated in underlined bold. Construct numbers correspond to lane numbers in B. **B**) Representative RT-PCR results following transfection of Yucatan fibroblasts with minigene constructs. Inclusion expressed as a percentage is indicated in the barplot, error bars indicate standard error of mean, n = 3. Lane numbers correspond to construct numbers in A.

We next examined if the splicing of *SMN2* exon 7 could be reestablished in a pig genomic context by introducing the exon 7+6 C>T mutation. First, we introduced the wild type pig sequence and a pig sequence with a mutation corresponding to the human +6C>T *SMN2* mutation into the pSXN13 splicing reporter as previously described [19], [23], [24] and transfected Yucatan pig fibroblasts with these constructs and constructs harboring the corresponding human sequences (Fig. 2A). This showed that the pig ESE sequence has splicing enhancer function, which is slightly weaker than the corresponding human ESE sequence, and that these ESEs are functional in pig cells. We then introduced a change corresponding to the human +6C>T *SMN2* mutation in the pig ESE sequence and observed that it decreased inclusion of the test exon, similar to the human *SMN2* sequence (Fig. 2B). Next we disabled the previously described flanking ESS in the inserted sequences by introducing an A>C mutation [19]. These results show that the ESE in the wild type pig sequence, although functional, is slightly weaker than the ESE in the corresponding human *SMN1* ESE sequence. They also show that this ESE activity in the pig sequence is decreased in the mutated pig *SMN2*-like sequence. Overall, the splicing patterns of pSXN13 constructs containing the pig sequences are very similar to corresponding constructs containing the human sequences (Fig. 2B), but it appears that the pig ESE is slightly weaker than the human *SMN1* ESE, and that the negative effect of the +6C>T *SMN2* mutation is weaker when introduced into the pig sequence.

**Figure 3:**
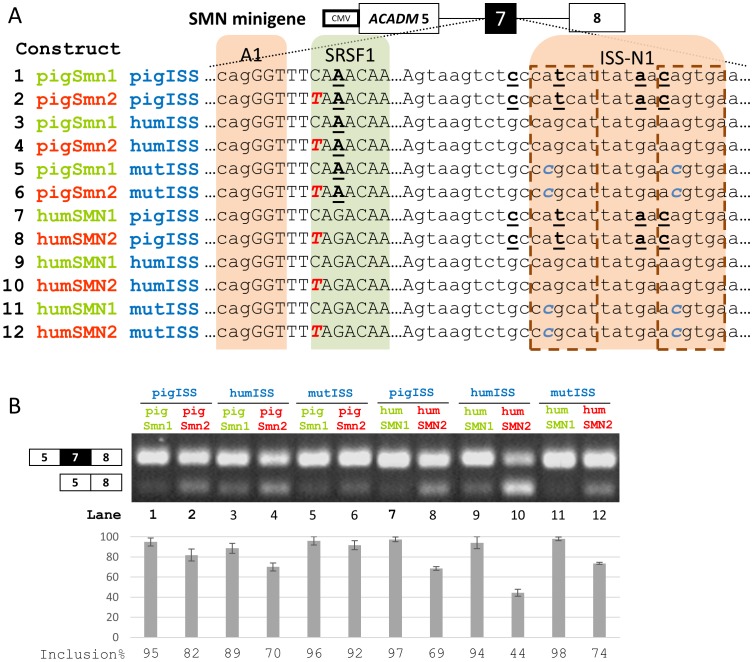
Splicing analysis of SMN minigenes. **A**) Summary of the SMN minigene and the mutations introduced in the different constructs. The hnRNP A1 binding sites within ISS-N1 have been indicated in dashed outline. Capitals indicate exonic bases. Bold underlined bases are bases that differ between humans and pigs. Bold italic bases in blue are mutations introduced. The +6C>T mutation is indicated in bold italic red. Dots within the sequence indicate a gap spanning multiple bases. Construct numbers correspond to lane numbers in B. **B**) RT-PCR results following transfection of Yucatan fibroblasts with minigene constructs. Inclusion expressed as a percentage is indicated in the barplot, error bars indicate standard error of mean, n = 3. Lane numbers correspond to construct numbers in A.

To investigate the splicing of pig *SMN* exon 7, we introduced the pig ESE motif into our *SMN* minigene [19] and replaced 25 bp of downstream intron 7 sequence with the corresponding pig sequence (Fig. 3A). The immediate upstream intron sequence containing the poly-pyrimidine tract (PPT) and 3′splice site (3′ss) is completely identical between pig and human. When we introduced the +6C>T mutation into the pig sequence the increase in exon 7 skipping was modest (Fig. 3B) and we speculated that this could be explained by the fact, that the intron splicing silencer (ISS) sequence in pig intron 7 is different from the human ISS [25]. In humans, the ISS contains two potential hnRNP A1 binding motifs, a proximal cagcat sequence and a distal aagtga sequence, but one of these, the proximal, is abrogated in the pig (Fig. 1B, cagcat>catcat). The distal hnRNP A1 binding site seems to be strengthened in the pig sequence (aagtga>cagtga). Therefore we tested both the human ISS sequence and a mutated human ISS sequence where both potential hnRNP A1 sites are disrupted by 2A>C mutations, which have previously been shown to disrupt hnRNP A1 binding [25] (Fig 3A, constructs 3–6). Interestingly, insertion of the human ISS resulted in a modest increase in pig exon 7 exclusion (Fig. 3B), indicating that the human ISS sequence has a stronger negative effect on *SMN* exon 7 inclusion than the corresponding pig ISS sequence. Insertion of the the mutated human ISS resulted in a very modest decrease in pig *SMN* exon 7 exclusion indicating that the pig ISS has only a very modest ISS activity (Fig. 3B).

We investigated the pig ISS sequence further in our human *SMN* minigene containing the human *SMN1* and *SMN2* ESE sequences. Also in this context the pig ISS does not inhibit inclusion of *SMN2* exon 7 as strongly as the human ISS, and the splicing pattern of the constructs with the mutated human ISS are indistinguishable from those with the pig ISS, indicating that the pig ISS is nearly inactive (Fig. 3B).

**Figure 4:**
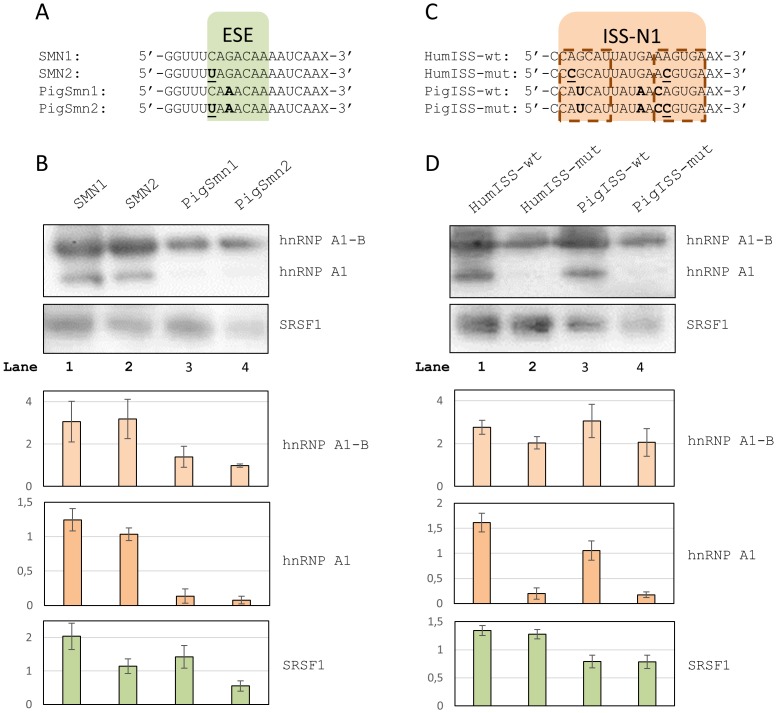
RNA affinity pull-down experiments. **A**) RNA oligos spanning the ESE in *SMN1* exon 7. Bases in bold indicate positions where the pig and the human sequences differ. The +6C>T mutation is indicated in bold underline. X indicates biotin. **B**) Western blots of protein pull-downs with oligos spanning the ESE. Two bands are seen for hnRNP A1, the upper band most likely being the alternative B splice isoform. Barplots indicate normalized band intensities for the indicated protein bands. Error bars indicate standard error of mean (n = 3). **C**) RNA oligos spanning the ISS in *SMN1* intron 7. Bases in bold indicate positions where the pig sequence differs from the human. Mutations introduced are indicated in bold underline. X indicates biotin. The hnRNP A1 binding sites within ISS-N1 have been indicated in dashed outline. **D**) Western blots of protein pull-downs with oligos spanning the ISS. Barplots indicate normalized band intensities for the indicated protein bands. Error bars indicate standard error of mean (n = 3).

To establish the interactions of the pig ESE and ISS with proteins known to bind the corresponding motifs in humans, we performed RNA-affinity purification experiments using HeLa nuclear extracts and RNA oligonucleotides with the sequences that harbor the wild type pig and human ESE and ISS motifs as well as mutated versions (Fig. 4A).

When we examined the binding of hnRNP A1 and SRSF1 to the ESE motifs, we observed binding of SRSF1 to the pig (*Smn1*-like) ESE, and much less binding of hnRNP A1 (Fig. 4B). The binding of hnRNP A1 to the mutated (*Smn2*-like) pig ESE was only slightly increased, indicating that reduced hnRNP A1 binding does not explain why the pig ESE retains some functionality when the +6C>T mutation is introduced. Importantly, the +6C>T mutated pig ESE bound SRSF1 very poorly, indicating that the loss of ESE activity observed in the PSXN13 construct could be due to loss of SRSF1 affinity (Fig. 4B). Similarly, we observed a decrease in binding affinity towards SRSF1 in the *SMN2* (+6C>T) construct relative to *SMN1*. These results therefore indicate that the SRSF1-binding ESE in human *SMN1* exon 7 is retained in pig and several other species with an identical motif (Fig. S2).

*In silico* analysis of the wild type pig ESE sequence versus the mutant using the Human Splicing Finder [26] (Table 1 and Fig. S3) revealed that an ESE element in the wild type pig *Smn1*-like sequence was removed according to ESEfinder which estimated a drop in SRSF1 and SRSF2 score to below threshold in the *Smn2*-like ESE, indicating that SRSF1 and SRSF2 are proteins likely to bind the wild type pig *Smn1* ESE motif but not to the mutant *Smn2*-like motif [27], [28]. The RESCUE-ESE algorithm similarly indicated loss of an ESE motif [29] and the PESE algorithm also reported a drop in score to below threshold [30], [31]. Additionally, new ESS motifs appeared in the mutant sequence according to the FAS-ESS algorithm [32] and the IIE algorithm [33].

**Table 1:** *In silico* analysis results.

ESE/ESS algorithm	Sequence	Position in exon 7	+6C score	+6T score
SRSF1 (ESEfinder)	CAAACAA	6	1.86	−1.23
SRSF2 (ESEfinder)	GGTTTCA	1	3.42	1.69
RESCUE-ESE	CAAACA	6	Site present	Not present
PESE (new)	GGTTTCAA	1	4.25	Below threshold
Fas-ESS	GGTTTT	1	Not present	Site present
IIE	GTTTTA	2	Not present	Site present
IIE	TTTTAA	3	Not present	Site present

The *in silico* analysis results of the wild-type pig ESE (+6C, CAAACAA) and the *Smn2*-like mutation (+6T, TAAACAA).

Using an intronic sequence spanning the full ISS motif as bait (Fig. 4C) we observed strong binding of hnRNP A1 to the human wild type ISS and reduced binding when the human ISS is mutated in both hnRNP A1 sites (Fig.4D), in agreement with earlier findings [25]. The wild type pig ISS on the other hand, displayed affinity towards hnRNP A1 to only a slightly lower degree than the human wild type ISS. A mutation abrogating the distal hnRNP A1 site in the pig ISS resulted in greatly reduced binding of hnRNP A1, indicating that hnRNP A1 binding to the pig ISS is mediated by the distal site (Fig 4D).

## Discussion

Initially, we sequenced the porcine *Smn1* gene from the Yucatan minipig, focusing on exonic sequences and intronic sequences surrounding the exons, and in particular exon 7. Others have previously published the sequence of the porcine *Smn1* gene [20] and the *Sus scrofa* genome is now available, but these sequences did not originate from the Yucatan subspecies. We therefore sequenced the Yucatan *Smn1* exons and parts of intron 6 and intron 7 in which we planned to position homology arms in order to edit the endogenous *Smn1* exon 7. This was prompted by the observation that even small differences between different subspecies can affect the efficiency of homologous recombination [34].

We observed that in agreement with the Sus scrofa reference genome, the pig *Smn1* gene contains a small 26 bp exon 8 which differentiates it from both the human *SMN1* gene and the murine *Smn1* gene. It is likely that the exon was activated by accumulation of mutations within intron 7 of the ancestral *Smn1* in the pig genome since this exon has not been observed in other species, but other scenarios may also explain why pig *Smn1* has an extra exon compared to humans. Since the exon is positioned downstream of the reading frame, and short enough for the NMD pathway not to be activated, it doesn't impact the expression of the SMN protein, but it is possible that it could lead to differences in the inclusion of *Smn1* exon 7 in the pig compared to humans and mice. We have not addressed this scenario in this study, but consider it unlikely that this exon would affect inclusion of exon 7 in a significantly negative way as pigs, like mice, only have one SMN producing gene. It is more likely that the greatly increased length of intron 7 in pigs would increase inclusion of exon 7 as it would delay the competition between exon 7 and exon 8, giving the spliceosome more time to recognize and process exon 7. This hypothesis is supported by previous studies showing that inhibition of exon 8 splicing enhances inclusion of *SMN2* exon 7 [35]–[37].

Another possibility is that pig exon 8 contains regulatory motifs such as miRNA binding sites. However, we consider this possibility unlikely since human *SMN1* and mouse *Smn1* transcripts have very dissimilar 3′UTR regions indicating that miRNA regulation through motifs located in the 3′UTR is unlikely to be involved in the regulation of SMN expression.

The ESE motif in pig *Smn1* exon 7 is slightly altered when compared to the human motif but is identical to the ESE motif found in other species such as cat and dog (Fig. S2). Notably, this ESE motif does not contain a G nucleotide and should, in theory, not constitute an hnRNP A1 binding site even when a +6 C>T mutation is introduced in exon 7. Our results support this hypothesis as we observed very low binding of hnRNP A1 to the pig ESE motif compared to the human ESE motif.

In pig *Smn1* intron 7 we also observed several differences in the sequence compared to humans, in particular, the previously identified ISS [25], [38] contained several substitutions that might lead to altered function when compared to the human ISS.

We first investigated the ESE potential of the CAAACAA sequence found in pig *Smn1* exon 7 to evaluate the feasibility of constructing a pig *Smn2-like* model by introducing a single +6C>T mutation in the endogenous porcine *Smn1* gene.

We introduced the ESE region into the pSXN13 splicing reporter minigene and observed minimal inclusion of the alternative exon in the wild type construct and complete loss of inclusion when we introduced a C>T mutation analogous to the +6T in *SMN2*. This demonstrates splicing enhancer activity of the altered pig ESE and further demonstrates that a C>T mutation can abrogate this activity despite the lack of an AG dinucleotide within the ESE which has previously been proposed to form an hnRNP A1 ESS in *SMN2* exon 7. Therefore, in this context the pig ESE supports the ESE-loss model. When we disrupted the upstream ESS motif by an A>C mutation, the splicing pattern of the wild type pig ESE and mutant pig ESE was very similar to that of *SMN1* and *SMN2*. However, the inclusion level of the wild type pig *Smn1*-like ESE construct seemed slightly lower than that of the wild type *SMN1* construct, while the inclusion level of the mutant pig *Smn2*-like ESE construct seemed slightly higher than that of the *SMN2* construct. These results can be explained by the ESE-loss/ESS-gain model. The G>A change in the pig ESE seems to have decreased the ESE activity relative to the human *SMN1* ESE sequence, but in the context of the +6C>T mutation, which completely abolishes the human *SMN1* ESE activity, the activity of the gained ESS has also been decreased. Overall, the regulatory element is more neutral in the pig *Smn1* gene than in human *SMN1* and *SMN2*, although it retains some ESE activity.

When we introduced pig *Smn1* exon 7 with flanking regions in our SMN model minigene, we observed only a modest decrease in exon inclusion between the construct with the wild type sequence and the +6C>T mutant construct (Fig. 3B), indicating that in the native pig *Smn1* gene a functional ESE is not crucial for exon 7 inclusion.

We then examined pig intron 7 and found that in the region of the previously reported IVS7-ISS [25], there were differences between the human and pig sequence which could potentially alter binding affinity of one or both of the hnRNP A1 sites contained within the ISS motif. In fact, the motif score was decreased for the proximal hnRNP A1 site and increased for the distal site through an A>C mutation which has previously been shown to increase skipping of human *SMN2* exon 7 [25].

These findings then lead us to investigate the ISS activity of the downstream region in pig *Smn1* intron 7 and we found that when we inserted the human ISS into the pig context, a splicing pattern more similar to *SMN2* splicing pattern was observed when we introduced the +6C>T mutation into the pig ESE sequence (Fig. 3B). When we introduced mutations previously reported to remove ISS activity [25], we observed exon inclusion similar to the wild type pig construct (Fig. 3B). It seems that the inhibitory function of the ISS is mostly dependent on the proximal hnRNP A1 site and despite strengthening of the distal hnRNP A1 site in the pig ISS, it is not enough to overcome the loss of the proximal site.

When we examined the function of *SMN1* and *SMN2* ESE sequences, we observed that the shift in exon inclusion was more pronounced between *SMN1* and *SMN2* than the equivalent +6C>T mutation in the pig ESE. One possible explanation is that the mutated pig ESE still retains some ESE activity whereas the *SMN2* ESE is completely inactivated. Another explanation could be that because the pig ESE does not have a central G it is not able to function as a low-affinity hnRNP A1 site and therefore it does not contribute negatively to the inclusion of exon 7 to the same extent as the inactivated *SMN2* ESE.

To determine whether the mutated pig ESE binds SRSF1 more efficiently than the *SMN2* ESE and if it binds hnRNP A1 less efficiently, we performed RNA-affinity purification experiments with RNA oligonucleotides spanning the ESE region. We observed strong binding of SRSF1 to the ESE motifs and much less to the mutated motifs in both pig and human context indicating that SRSF1 may enhance splicing of both human *SMN1* and porcine *Smn1*, but also that the residual ESE activity of the mutated pig ESE may be through the binding to another SR protein. There was also diminished hnRNP A1 binding to both the wild type pig ESE and the mutated pig ESE. The reason is likely that the SRSF1-binding ESE region in pig *Smn1* constitutes an AC rich element. These elements have previously been shown to function as ESEs [39] and they do not contain any AG di-nucleotides, which we and others have demonstrated to be essential for efficient hnRNP A1 binding [19], [40], [41].

*In silico* analysis identified SRSF1 and SRSF2 as possible proteins binding to an ESE in the pig wild type sequence, but not the mutant sequence. However, since the SRSF2 motif and the 3′ splice site are juxtaposed, it is not likely to be acting as a splice enhancer if it is indeed functional. Although we did not observe binding of SRSF1 to the mutated pig ESE at a level comparable to the SMN1 ESE, this construct is artificial and although it may have residual ESE activity through binding to another SR protein, it does not necessarily follow that this SR protein is binding to the natural pig ESE *in vivo*. SRSF1 therefore remains a likely candidate as the SR protein binding to the ESE motif in pigs as well.

While the *in silico* analysis indicated that the porcine ESE activity was diminished when the ESE was mutated to an *Smn2*-like motif (Table 1), we did not observe a pronounced increase in exon skipping in the mutant constructs. In the pSXN13 reporter minigene constructs, however, the pig ESE behaved almost identically to the human ESE indicating that some ESE activity is indeed lost. These results indicate that in the context of pig *Smn1* exon 7, a strong ESE at position +6 is not a requirement for efficient splicing, but it may still contribute to a stronger definition of the exon.

The RNA-protein affinity studies of the IVS7-ISS pig motif revealed overall similar binding of hnRNP A1 to the motif, compared to the human wild type IVS7-ISS, despite a putative increase in the strength of the distal hnRNP A1 core motif. This is most likely caused by the disruption of the proximal motif which may result in a complete loss of hnRNP A1 binding to that site. The loss of this site is likely enough to effectively disrupt the function of the entire ISS in the context of pig *Smn1* exon 7. This is in line with previous evidence that suggests a more central role of the proximal site [25] compared to the distal site. In the context of human *SMN2* exon 7, abrogation of this site improves the inclusion of exon 7, indicating that this site is a major contributor to the inefficient splicing of human *SMN2* exon 7. Additionally, the IVS7-ISS has proved a valuable therapeutic target and has resulted in on-going clinical trials with an ASO specifically blocking this ISS motif [16], [17], [42].

Together, these findings indicate that in pigs, the IVS7-ISS is inactive due to a mutation in the proximal hnRNP A1 site and this inactivation has relaxed the requirements for a strong ESE at the 3′ss of exon 7. This indicates that insertion of an *SMN2*-like mutation in the endogenous porcine *Smn1* gene would be insufficient to recapitulate the splicing of *SMN2* exon 7 and that insertion of a larger fragment of the human *SMN2* gene would be required.

In the mouse, the SRSF1 ESE is identical to the human ESE but like the pig, the mouse IVS7-ISS is abrogated in the proximal site (Fig. 1B). Furthermore, the distal site does not appear to be strengthened indicating that the ISS is likely to be functionally weakened also in the mouse. These observations indicate that the human ISS would have to be inserted into the murine *Smn1* gene if *Smn1* exon 7 skipping was to be induced by an +6C>T mutation, but a mouse model containing a murine *Smn1* to *Smn2*-like conversion has been published without insertion of the human ISS [43]. This model exhibited a milder SMA phenotype similar to human SMA type III, indicating that even though the *Smn2*-like exon 7 was skipped, the level of inclusion was still higher than human *SMN2* exon 7, resulting in a mild phenotype. One possible explanation for this, is that the murine ISS contains a 3 bp deletion which moves the distal site in closer proximity to the 5′ss, although not as close as the proximal site of the human ISS. This indicates that the inhibitory function of this hnRNP A1 core motif may be highly dependent on its proximity to the 5′ss and that it may function by directly blocking access of U1 snRNP to the 5′ss, and not by inducing a polymerization of hnRNP A1 proteins along exon 7, or by looping out the exon through interaction with other ISS/ESS elements, such as the hnRNP A1 binding motif spanning the 3′ss of exon 7 or the hnRNP A1 motif generated across the previous SRSF1 binding ESE in *SMN2* [18], [19].

In conclusion, we established the presence of a functional and likely SRSF1 binding ESE in porcine *Smn1*, that this ESE may be disrupted by a mutation similar to the +6C>T transition found in *SMN2* exon 7, and that porcine *Smn1* is not dependent on activity of this ESE due to the absence of a functional ISS motif in the region immediately down-stream of porcine *Smn1* exon 7. These findings illustrate how key regulatory motifs may be switched on and off in a specific order and that ESE motifs may be more evolutionary conserved than ISS motifs, particularly in the context of indispensable exons.

Furthermore, we found that splicing of porcine *Smn1* is similar to *SMN1* in many ways, but that the regulation also differs significantly. Crucially, the highly relevant therapeutic target region containing ISS-N1 is disrupted in pigs and even if skipping of porcine *Smn1* could be induced by a single mutation, therapeutics targeting this ISS would not be efficacious in this model.

We therefore suggest that porcine *Smn1* may be converted to a *Smn2*-like gene through insertion of a region spanning the end of intron 6, the full exon 7 and intron 7, and the beginning of exon 8 of human *SMN2* via techniques such as homologous recombination. This would produce a hybrid SMN protein with a slightly different C-terminal sequence derived from the human exon 7, but we do not believe that this would significantly impact protein function, as the C-terminal has previously been demonstrated to be less dependent on the exact amino-acid sequence [44].

## Materials and Methods

### Minigene constructs

The minigenes used in this study were based on the *SMN* human minigene described previously [19] and the pSXN13 splicing reporter minigene also previously described [19], [23], [24].

*SMN* minigenes. All *SMN* minigenes were ordered from and prepared by Genscript (Piscataway, NJ, USA).

*pSXN13* minigenes. To generate pSXN13 constructs we used sense and antisense oligonucleotides with desired sequences, which were mixed 1∶1, phosphorylated and ligated to the BamHI and SalI sites in the artificial exon within pSXN13 [24]. The sequences of the generated plasmids were verified by DNA sequencing.

### Transient transfection of Yucatan fibroblasts and splicing analysis

App. 2.0×10^5^ Yucatan fibroblasts were seeded in 6 well plates and the following day transfected with 0.8 µg expression plasmid, either *SMN* constructs or pSXN13 constructs, using FuGENE6 transfection reagent (Roche, Mannheim, Germany). Transfections were performed in biological triplicates using fibroblasts from Yucatan minipigs. 48 hours post transfection cells were washed in 1 x PBS-EDTA, lysed by adding 900 µL TRIzol reagent (Invitrogen Co., Carlsbad, CA) and incubated on ice for 10 min prior to RNA extraction according to the manufacturer's instructions (Invitrogen). Purified total RNA was used as template in first strand cDNA synthesis using the Advantage MMLV RT-PCR kit (BD Biosciences Clontech, Franklin Lakes, NJ) with an oligo (dT)_18_ primer. App. 1/10 of the cDNA synthesis product corresponding to 100 ng RNA was used as template in each PCR reaction using Tempase DNA polymerase (Ampliqon Aps, Skovlunde, Denmark). For the *SMN* constructs we used an exon-exon junction spanning primer (CATTCCAGAGAACTGTGGAGGT) and a primer located in *SMN1/2* exon 8 (GTGGTGTCATTTAGTGCTGCTC). For the pSXN13 constructs we used a primer located in β-actin exon 1 (AAGGTGAACGTGGATGAAGTTGGTGGTG) and an exon-exon junction spanning primer (CCCACGTGCAGCCTTTGACCTAGTA). PCR products were separated and visualized on agarose gels containing ethidium bromide (EtBr) on an Epi II Darkroom UVP Transilluminator. Bands were quantitated by optical densitometry using ImageJ 1.47 [45] and normalized to the length of the PCR product. Subsequently inclusion percentage was estimated as the normalized intensity of the upper band divided by the sum of the normalized intensities of the upper and lower band.

### Amplification and sequencing of Yucatan minipig genomic DNA

Genomic DNA from Yucatan fibroblasts was extracted and 40 ng used as template in PCR reactions using Pfu polymerase (Promega, Madison, WI, USA) with primers listed in table S1.

Amplified PCR products were sequenced on the ABI Prism 3100-Avant (Applied Biosystems, Foster City, CA). The partially complete Yacatan minipig *Smn1* sequence was submitted to GenBank database under accession number KF585502.

### RACE experiments

The 5′RACE and 3′RACE experiments were carried out according to the manufacturer's instructions using the SMARTer RACE kit (BD Biosciences Clontech).

### Protein pull down by biotin coupled RNA oligonucleotides

We used 3′ biotinylated RNA oligonucleotides spanning either the ESE motif: *SMN1*: 5′-GGUUUCAGACAAAAUCAA-biotin, *SMN2*: 5′-GGUUUUAGACAAAAUCAA-biotin, pigSmn1: 5′-GGUUUCAAACAAAAUCAA-biotin, pigSmn2: 5′-GGUUUUAAACAAAAUCAA-biotin or the ISS motif: ISShum wt: 5′-CCAGCATTATGAAAGTGAA-biotin, ISShum mut: 5′-CCCGCATTATGAACGTGAA-biotin, ISSpig wt: 5′-CCATCATTATAACAGTGAA-biotin, ISSpig mut: 5′-CCATCATTATAACCGTGAA-biotin. Pull-down assays and immunodetection of purified proteins were carried out as previously described [40] in three separate experiments using three separate nuclear extracts. For each purification, 100 pmol of RNA oligonucleotide was coupled to 100 µl of streptavidin-coupled magnetic beads (Dynal) for 15 min in 1xbinding buffer (20 mM Hepes/KOH [pH 7.9], 72 mM KCl, 1.5 mM MgCl, 1.56 mM MgAc, 0.5 mM DTT, 4 mM glycerol, 0.75 mM ATP, and 0.2 µg/µl bulk tRNA). The suspension was then placed in the magnet, and the supernatant removed. The oligonucleotide-bead complexes were then resuspended in 500 µl 1xbinding buffer containing 100 µL nuclear extract purified from HeLa cells over-expressing either hnRNP A1 or SRSF1 and incubated 25 min at room temperature. Then, the supernatant was removed, and the beads were washed three times in 500 µl 1xbinding buffer containing 300 mM KCl. Finally, the proteins bound to the RNA were eluted by the addition of 50 µl protein sample buffer and heating for 4 min at 90°C. Subsequently, 12 µl of the protein eluates were run on a 4–15% SDS-gel and western blotted using mon-clonal antibodies against hnRNP A1 (R9778, Sigma-Aldrich, Saint Louis, MO, USA) and SRSF1 (32–4500, Invitrogen). Bands were quantitated by optical densitometry using ImageJ 1.47 [45] and normalized to the geometric mean band intensity per protein.

### In silico analysis

We used Human Splicing Finder [26] to examine the sequences for ESEs [27]–[31], [33] and ESSs [30]–[33], [46]. Wild-type and mutant sequence covering the last 3 bp of intron 6 and the first 15 bp of exon 7 were submitted using mutation analysis as the selected analysis mode. Sequence alignments were done using MAFFT [47]. *SMN1* exon 7 orthologue sequences were extracted from the Ensembl database including 20 bp upstream and 30 bp downstream of exon 7.

## Supporting Information

Figure S1 Alignment of the Sus scrofa *Smn1* mRNA and the Sus scrofa *Smn1*-pseudogenes. Capitals in the *Smn1* sequence indicate the SMN open reading frame, dashes indicate gaps in the alignment.
(DOCX)

Figure S2 Multiple alignment of *SMN1* exon 7 and mammalian orthologues. MAFFT multiple alignment of *SMN1* exon 7 with spanning intronic sequences and seven mammalian orthologues. Capitals indicate exonic bases, dashes indicate gaps. Asterisks indicate fully conserved bases while periods indicate partially conserved bases.
(DOCX)

Figure S3 Analysis of human *SMN1* and porcine *Smn1*. Graphical output of analysis of the ESE region in human *SMN1* and porcine *Smn1* using Human Splicing Finder. A drop in ESE strength is indicated, as well as gain of a putative ESS in the context of porcine *Smn1* relative to human *SMN1*.
(DOCX)

Table S1 Primers used for amplification of the porcine *Smn1* gene from genomic DNA from Yucatan fibroblast. The listed primers were used for PCR amplification using genomic DNA from Yucatan fibroblasts as template.
(DOCX)
