# Supplementary figures and images for: Absence of an Intron Splicing Silencer in Porcine Smn1 Intron 7 Confers Immunity to the Exon Skipping Mutation in Human SMN2
Source: PLoS One. 2014 Jun 3;9(6):e98841. doi: 10.1371/journal.pone.0098841 (PMC4043917; doi:10.1371/journal.pone.0098841)

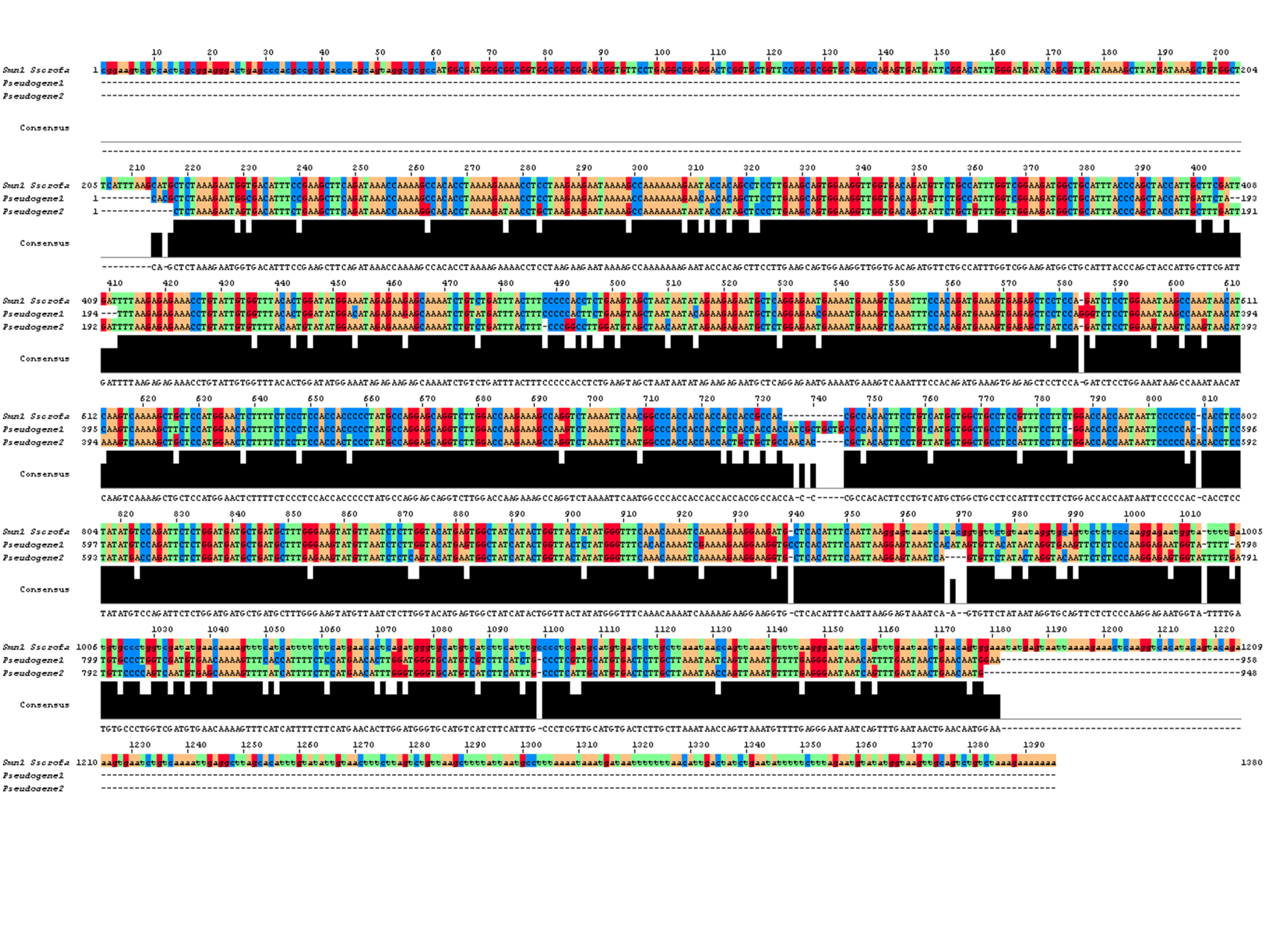

Supplement: Figure S1 — Alignment of the Sus scrofa Smn1 mRNA and the Sus scrofa Smn1-pseudogenes. Capitals in the Smn1 sequence indicate the SMN open reading frame, dashes indicate gaps in the alignment. (DOCX) [file pone.0098841.s001.docx]

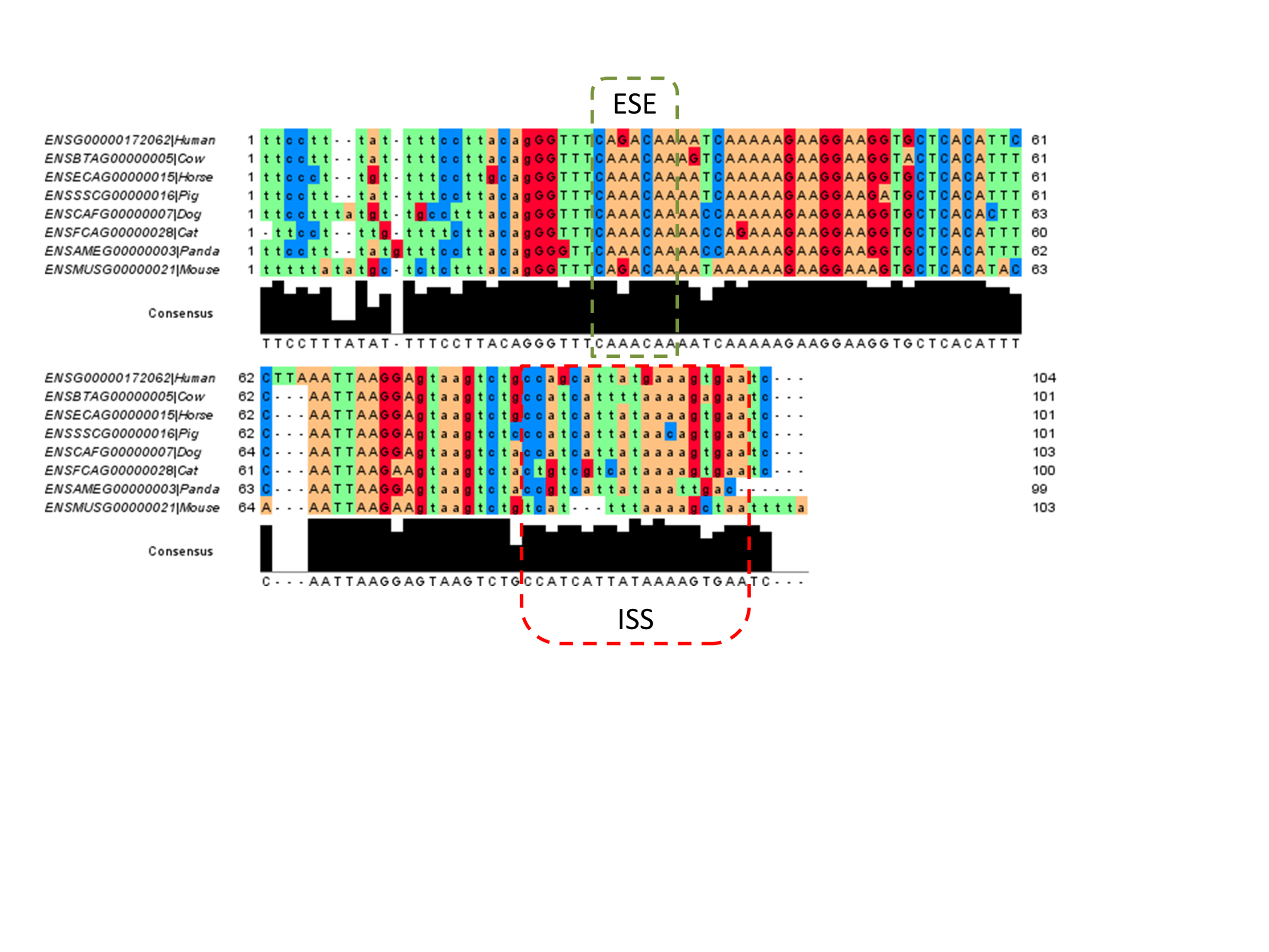

Supplement: Figure S2 — Multiple alignment of SMN1 exon 7 and mammalian orthologues. MAFFT multiple alignment of SMN1 exon 7 with spanning intronic sequences and seven mammalian orthologues. Capitals indicate exonic bases, dashes indicate gaps. Asterisks indicate fully conserved bases while periods indicate partially conserved bases. (DOCX) [file pone.0098841.s002.docx]

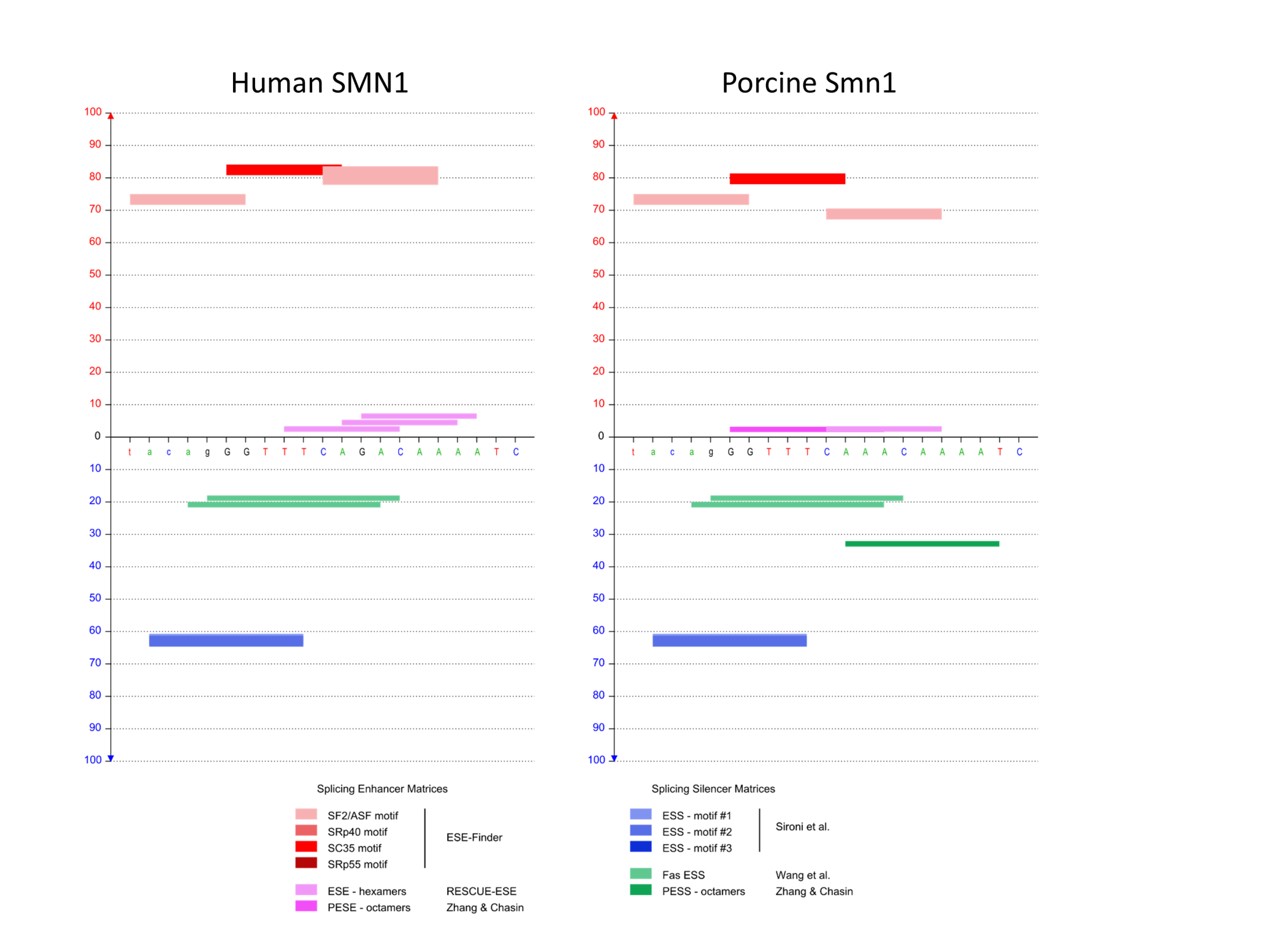

Supplement: Figure S3 — Analysis of human SMN1 and porcine Smn1. Graphical output of analysis of the ESE region in human SMN1 and porcine Smn1 using Human Splicing Finder. A drop in ESE strength is indicated, as well as gain of a putative ESS in the context of porcine Smn1 relative to human SMN1. (DOCX) [file pone.0098841.s003.docx]
