# Supplementary material for: Absence of an Intron Splicing Silencer in Porcine Smn1 Intron 7 Confers Immunity to the Exon Skipping Mutation in Human SMN2
Source: PLoS One. 2014 Jun 3;9(6):e98841. doi: 10.1371/journal.pone.0098841 (PMC4043917; doi:10.1371/journal.pone.0098841)
Supplement: Table S1 — Primers used for amplification of the porcine Smn1 gene from genomic DNA from Yucatan fibroblast. The listed primers were used for PCR amplification using genomic DNA from Yucatan fibroblasts as template. (DOCX) [file pone.0098841.s004.docx]

**Table S1**

**Primers used for exon amplification**

SMNpig.ex1seq.s: 5’-AAGCGCTGGTTGTTACTCTCTCCAGC-3’

SMNpig.ex1seq.as: 5’-CTTAGAAACATGCCCACCACGACCTG-3’

SMNpig.ex2aseq.s: 5'-TGAGCAGGTAGGCTTCTTGTGGT-3'

SMNpig.ex2aseq.as: 5'-AGCAACAGTGGATCTGAGCTGCA-3'

SMNpig.ex2bseq.s: 5’-TGGACCTAGAGACCAGTTTATAGTGAC-3’

SMNpig.ex2bseq.as: 5’-ACATGTCAATGGAAAACACTGTAGTACTC-3’

SMNpig.ex3seq.s: 5’-TCCCAGAAAAGTTAACTGGAAGCTACTG-3’

SMNpig.ex3seq.as: 5’-GCCTCAGTGGATCAAACTAGTTATACC-3’

SMNpig.ex4seq.s: 5’-GGTATAACTAGTTTGATCCACTGAGGC-3’

SMNpig.ex4seq.as: 5’-CAATTCACTAACAAGGGGCTGAATGTC-3’

SMNpig.ex5seq.s: 5’-AGTGGGTACTCCTGTGTAGCCTTCTTG-3’

SMNpig.ex5seq.as: 5’-ACCTATGTTCTGCTAAATTCCAAACTGTG-3’

SMNpig.ex6seq.s: 5’-AAAGTAGGAGATGATCAGGAATGTAGG-3’

SMNpig.ex6seq.as: 5’-CTGACATACGCCTATAGTTCCCAATC-3’

SMNpig.ex8seq.s: 5’-GTGAGGGAAACTTCCCAGTGTACTC-3’

SMNpig.ex8seq.as: 5’-TGCAACTTGGAACATATGTGGCCAG-3’

SMNpig.ex9seq.s: 5’-TTGTATTTACTGCGACAAGTGTGTTGC-3’

SMNpig.ex9seq.as: 5’-CACAATCGGCTCTATCTTTACAACACTAC-3’

**Primers used to amplify the intronic regions surrounding exon 7**

SMN-Ss-ivs6.2-XhoI-s: 5’-CGGCATCTCGAGGTTCCCAGGCTAGGGGTCGAATTGGAGC-3’

SMNpig.ivs6-368.as: 5’-AGGTTGCGGGTTTGATCTCTGACC-3’

SMNpig.ivs6-177as: 5'-GTGATCCCAAAGTAAACAATAATTAGGCTGC-3'

SMN-Ss-Seq-ivs7-s: 5’-GTCTAATTACTTAACTGTCCCATAAAGTTTG-3’

SMN-Ss-Seq-ivs7-as: 5’-CAAACTTTATGGGACAGTTAAGTAATTAGAC-3’

SMNpig.ivs7.300s: 5'-GATAGTCAGACTTGCTTTTTGACTTGTGATG-3'

SMNpig.ivs7.586s: 5’-AAGAGATATAAGAGCCAGGGTGTTCC-3’

SMNpig.ivs7.658s: 5’-CAGGTTCAATCTCTGACCTCACTCAG-3’

SMNpig.ivs7.963s: 5’-TGTTGCTGATTTATGAGTTGTGG-3’

SMNpig.ivs7.1321s: 5’-TCCCCACTTAACATAGTCTTG-3’

SMNpig.ivs7.1635s: 5’-CTCAGATTGTTTGCCTGTGTGTC-3’

SMNpig.ivs7.1971s: 5’-CTCTGGCAAAAGATCGTAGACAC-3’

SMNpig.ivs7.2217s: 5’-TGCAATGACACTGGATTCTAAAC-3’

SMNpig.ivs7.2512s: 5’-CCCTAAAGTCATTTTTATTCAGTTGG-3’

SMNpig.ivs7.2632as: 5’-CTGCCCTAATGCTTCTACCTTTGTAC-3’

SMNpig.ivs7.2847s: 5’-GGGGGTCAGGTTATCCTTACATG-3’

SMNpig.ivs7.3195s: 5’-GTTGCTGCAAATGGCATTATGTC-3’

SMNpig.ivs7.3527s: 5’-GCCCCTCCAACACTTGTTATTTG-3’

SMNpig.ivs7.3890s: 5’-GTCCCATGGGTTTATTTTTGCTC-3’

SMNpig.ivs7.4234s: 5’-GGTCTGTCTGTCTGTTTTGGTAC-3’

SMNpig.ivs7.4560s: 5’-ATTAAAGTTTTGTAGTTCTCGGC-3’

SMNpig.ivs7.4914s: 5’-CGATGTTGAAGAGCAGTGGTGAG-3’

SMNpig.ivs7.5236s: 5’-TCGTCTTGTCTACAGTATCTGGC-3’

SMNpig.ivs7.5448s: 5’-GTGAATGCTTTTGAAAGTTGAATTAC-3’

SMNpig.ivs7.5562as: 5’-AGACATTGAGTTTGTGGTGACTTTGC-3’

SMNpig.ivs7.5642s: 5’-TAATTGTCCTTGCGGTTGTTAGG-3’

SMNpig.ivs7.5959s: 5’-GCCTTCATCAAAGGATCAGTCAG-3’

SMNpig.ivs7.6307s: 5’-TTGGCGTTTGTTATTTTTATGTG-3’

SMNpig.ivs7.8677s: 5’-GGTTACCTCTGAGGAGGGACTAGAAG-3’
